# Effects of β-Adrenergic Antagonists on Chemoradiation Therapy for Locally Advanced Non-Small Cell Lung Cancer

**DOI:** 10.3390/jcm8050575

**Published:** 2019-04-26

**Authors:** Kunal R. Chaudhary, Sherry X. Yan, Samuel P. Heilbroner, Joshua R. Sonett, Mark B. Stoopler, Catherine Shu, Balazs Halmos, Tony J.C. Wang, Tom K. Hei, Simon K. Cheng

**Affiliations:** 1 Department of Radiation Oncology, Vagelos College of Physicians & Surgeons, Columbia University Irving Medical Center, New York, NY 10032, USA; kc2849@cumc.columbia.edu (K.R.C.); sherry.yan@nyumc.org (S.X.Y.); sam.heilbroner@gmail.com (S.P.H.); tjw2117@cumc.columbia.edu (T.J.C.W.); 2 Division of Cardiac, Vascular and Thoracic Surgery, Department of Surgery, Vagelos College of Physicians & Surgeons, Columbia University Irving Medical Center, New York, NY 10032, USA; js2106@cumc.columbia.edu; 3 Herbert Irving Comprehensive Cancer Center, New York Presbyterian Hospital-Columbia University Irving Medical Center, New York, NY 10032, USA; mbs5@cumc.columbia.edu (M.B.S.); cas2145@cumc.columbia.edu (C.S.); bahalmos@montefiore.org (B.H.); 4 Division of Hematology/Oncology, Department of Medicine, Vagelos College of Physicians & Surgeons, Columbia University Irving Medical Center, New York, NY 10032, USA; 5 Department of Oncology, Albert Einstein College of Medicine of Yeshiva University/Montefiore Medical Center, Bronx, NY 10461, USA; 6 Center for Radiological Research, Vagelos College of Physicians and Surgeons, Columbia University, New York, NY 10032, USA; tkh1@cumc.columbia.edu; 7 Department of Environmental Health Sciences, Mailman School of Public Health, Columbia University, New York, NY 10032, USA

**Keywords:** beta-blocker, repurposed drugs, chemotherapy, radiation, sensitivity, non-small cell lung cancer

## Abstract

Introduction: Locally advanced non-small cell lung cancer (NSCLC) is highly resistant to chemoradiotherapy, and many cancer patients experience chronic stress. Studies that suggest stimulation of β-adrenergic receptors (β-AR) promotes tumor invasion and therapy resistance. We investigated whether β-AR inhibition with beta-blockers acts as a chemotherapy and radiation sensitizer in vitro and in patients treated with chemoradiation for locally advanced NSCLC. Methods: We investigated the effects of the non-selective beta-blocker propranolol on two human lung adenocarcinoma cell lines (PC9, A549) treated with radiation or cisplatin. We retrospectively evaluated 77 patients with Stage IIIA NSCLC who received induction chemoradiation followed by surgery. Pathological and imaging response, metastatic rate, and survival were analyzed using SPSS v22.0 and PrismGraphpad6. Results: Propranolol combined with radiation or cisplatin decreased clonogenic survival of PC9 and A549 cells in vitro (*p* < 0.05). Furthermore, propranolol decreased expression of phospho-protein kinase A (p-PKA), a β-adrenergic pathway downstream activation target, in both cell lines compared to irradiation or cisplatin alone (*p* < 0.05). In patients treated for Stage IIIA NSCLC, 16 took beta-blockers, and 61 did not. Beta-blockade is associated with a trend to improved overall survival (OS) at 1 year (81.3% vs 57.4%, *p* = 0.08) and distant metastasis-free survival (DMFS) (2.6 years vs. 1.3 years, *p* = 0.16). Although beta-blocker use was associated with decreased distant metastases (risk ratio (RR) 0.19; *p* = 0.03), it did not affect primary tumor pathological response (*p* = 0.40) or imaging response (*p* = 0.36). Conclusions: β-AR blockade enhanced radiation and cisplatin sensitivity of human lung cancer cells in vitro. Use of beta-blockers is associated with decreased distant metastases and potentially improved OS and DMFS. Additional studies are warranted to evaluate the role of beta-blockers as a chemoradiation sensitizer in locally advanced NSCLC.

## 1. Introduction

Concurrent chemoradiation therapy is a standard treatment for non-small cell lung cancer (NSCLC) patients with locally advanced disease. However, resistance to therapy still leads to a dismal overall survival of 25% at 5 years and local disease failure in up to 50% of these patients [1]. Significant effort has been dedicated to investigate chemotherapy and radiation sensitizers that enhance the therapeutic effect on tumors to improve local disease control and prolong survival. Beta-blockers, which form a Food and Drug Administration (FDA)-approved class of oral medications that competitively antagonize β-adrenergic receptors (β-AR), have been shown to have antitumor activity and thus appear as an attractive option as therapeutic sensitizers. 

A growing body of knowledge has contributed to the understanding of the role the sympathetic nervous system may play in oncogenesis and cancer progression. One possible link between smoking and cancer development is that nicotine and nicotine-derived compounds have a high affinity for β-adrenergic receptors, and nicotine increases proliferation of human lung adenocarcinoma in vitro [2,3,4]. Further investigation revealed that nicotine not only mediates tobacco-induced cancer growth through the nicotinic acetylcholine receptor, but it may also activate β-AR, resulting in enhanced mitogenic signaling [4]. Nicotine also stimulates the secretion of neurotropic factors epinephrine and norepinephrine, which in turn bind β-AR, leading to a host of downstream oncogenic signaling cascades including the epidermal growth factor receptor (EGFR)/phosphoinositide 3-kinase/protein kinase B (PI3K/AKT), and mitogen-activated protein kinase (MAPK also known as RAS/Raf/MEK/ERK) pathways [5,6,7,8,9]. 

Stimulation of the β-adrenergic pathway has been observed to affect cancer biology by promoting tumorigenesis, cellular proliferation, tumor invasion, and resistance to chemotherapy in various cell lines and tumor models [10,11,12,13,14,15,16,17,18,19]. Preclinical studies found that β-AR inhibition reduces tumor cell migration and chemotaxis in human breast and prostate cancer cell lines [20,21]. In an orthotopic mouse model of breast cancer, the administration of propranolol, a non-selective β-AR antagonist, blocked distant tumor metastasis in vivo [18]. Other preclinical studies have revealed that activation of β-AR by adrenergic agonists stimulated tumor proliferation and angiogenesis, which may be reversed by β-AR inhibition [22,23]. Experiments with NSCLC lines also demonstrated β-AR inhibition reduced downstream pathway signaling and cell proliferation [24]. Interestingly, activation of β-AR by temperature-induced stress led to chemotherapy resistance in a pancreatic tumor model [19]. In a study by Liao et al., human gastric adenocarcinoma cell lines treated with propranolol in addition to radiation demonstrated enhanced radiation-induced cell killing. Protein expression analyses suggested that the radiosensitizing effects may have be mediated by inhibition of the beta-adrenergic pathway and downstream nuclear factor kappa-light-chain-enhancer of activated B cells (NF-κB)/EGFR/cyclooxygenase-II (COX-2) signaling [25].

Similarly, retrospective clinical reviews observed that incidental concurrent use of beta-blockers, a β-AR antagonist commonly taken for hypertension and heart failure, increased relapse-free survival and overall survival, reduced cancer-specific mortality and disease recurrence in patients treated for melanoma, breast and prostate cancers [26,27,28]. One group at the MD Anderson Cancer Center also identified beta-blocker use as an independent factor associated with improved survival in a large cohort of NSCLC patients treated with definitive radiation therapy [29,30]. Cancer patients experience increased stress associated with cancer diagnosis and treatment. If hormones involved in chronic stress promote treatment resistance in NSCLC, then beta-blockers may decrease the pro-oncogenic effect of beta-adrenergic mediated downstream signaling. 

However, the exact mechanisms of how beta-blockers confer their antitumor activity in lung cancers have yet to be understood. The use of this class of medication as a sensitizer in treatment of locally advanced NSCLC with concurrent chemoradiation remains to be further explored. We investigated whether β-AR inhibition with beta-adrenergic receptor antagonists, or beta-blockers, results in increased cisplatin and radiation sensitivities in vitro and differential treatment outcomes in patients treated with chemoradiation followed by surgery for locally advanced stage IIIA NSCLC. We hypothesized that beta-blockers increase cisplatin and radiation sensitivity of human lung cancer cells, and beta-blocker use confers survival benefit in patients treated for locally advanced NSCLC.

## 2. Materials and Methods

### 2.1. Cell Culture and Treatment

Two human lung adenocarcinoma cell lines, PC9 and A549, were obtained from the ATCC. Cells were cultured in complete RPMI 1640 medium supplemented with 10% fetal bovine serum and antibiotics. Cells were maintained at 37 °C with 5% CO_2_ in a humidified incubator. When cells reached logarithmic growth, they were split and allowed to attach overnight followed by treatment with the non-specific β-AR antagonist propranolol (1 μM and 50 μM) and radiation (0, 2, 4, 6, 8, 10 gray (Gy)) or cisplatin (0.2, 0.5, 1.0 μM). Propranolol and cisplatin were obtained from Sigma (St. Louis, MO, USA). Cell irradiation was performed with a Gammacell 40 Cesium irradiator (Atomic Energy of Canada, Ottawa, Canada) at a dose rate of 1 Gy/min. 

### 2.2. Cell Survival Analysis

To evaluate the clonogenic potential of PC9 and A549 cells after treatment, cells were plated in six-well plates and treated with cisplatin or radiation. Colonies were fixed with methanol and stained with Giemsa. The number of colonies containing at least 50 cells was determined, and the plating efficiency and surviving fractions were calculated as previously described [31]. Dose-survival curves were plotted. The clonogenic assay was performed three times, with triplicate plates for each experiment.

### 2.3. Western Blot Analysis

PC9 and A549 cells were pre-incubated with propranolol (50 μM) for 8 h and then treated with either radiation (6 Gy) or cisplatin (0.5 μM). Protein expression analyses were performed 4 h post-treatment for phospho-gamma nucleosome core histone H2A (phospho-γH2AX) and 24 h post-treatment for phopho-protein kinase A (phospho-PKA C). Cell lysates were prepared from with 4 × LDS sample buffer and resolved on SDS-PAGE according to standard protocols. Primary antibodies used included polyclonal anti-phospho-PKA C (Thr197) and anti-phospho-γH2AX (Cell Signaling, Beverly, MA, USA). The secondary antibodies (anti-rabbit or anti-mouse) were conjugated with horseradish peroxidase. Signals were detected using the ECL system (Amersham, Pittsburgh, PA, USA). Using ImageJ software, densitometries of phospho-protein kinase A (p-PKA) and p-γH2AX protein levels were quantified by normalizing over the vinculin, which served as a loading control. 

### 2.4. Lung Cancer Patient Cohort

Patients in this retrospective review were identified from an institutional review board-approved clinical database of NSCLC patients, who were treated at the New York Presbyterian-Columbia University Medical Center from 2005 to 2012. Patients with pathologically proven stage IIIA (per American Joint Committee on Cancer (AJCC) “7th edition”) NSCLC treated with neoadjuvant platinum-based chemoradiation therapy followed by surgery were included in this analysis. We chose to include only patients treated with this unique trimodality approach because pathological analysis of tumor response to chemoradiation would be assessable. All patients were staged using computed tomography of the chest, imaging of the brain, and positron emission tomography (PET). Pathologic or radiological mediastinal evaluation was required prior to treatment. All patients underwent pulmonary function testing. Each case was discussed at a multidisciplinary tumor board consisting of medical oncology, radiation oncology, and thoracic surgery. Our institutional approach for neoadjuvant radiation for locally advanced NSCLC is 45 to 60 Gy in 1.8 to 2 Gy fractions. If normal tissue constraints were not achieved, the dose was de-escalated to as low as 41.4 Gy. Concurrent chemotherapy was a cisplatin-based doublet therapy. Surgery is routinely performed within two months of completion of induction chemoradiation. 

### 2.5. Study Variables and Outcomes

Patient demographics, co-morbidities, tumor radiographic and histologic details, complete treatment data, and survival and recurrence data during long-term follow up were collected. Patient medical and pharmacy records were reviewed for the use of beta-blockers (nonspecific β antagonists or β-1 specific antagonists) throughout the course of their cancer treatment. The use of other medications, including aspirin and angiotensin-converting enzyme inhibitors (ACEi), which may confound the analysis of beta-blockers, was tabulated. Patients who were taking beta-blockers at the beginning of their neoadjuvant cancer therapy were included in the beta-blocker group. Local primary tumor pathological and imaging response, distant metastasis, distant-metastasis-free survival (DMFS), progression-free survival (PFS), and overall survival (OS) were analyzed. 

Primary tumor response was assessed by comparing the largest diameter of the lung tumor on baseline CT scans with that on CT scans 4–6 weeks after chemoradiation. Primary tumor pathological response rate was determined from the percent residual viable tumor at time of surgery. DMFS was defined as the time from the date of surgery to the date of first documented distant metastasis. PFS was measured from the date of surgery to the date of first documented locoregional recurrence or distant metastasis. OS was defined as time from the date of surgery to the date of last follow up or date of death. Patients who were lost to follow up or died without disease recurrence were censored at the date of last follow up or death. 

### 2.6. Statistical Analysis

Primary tumor pathological and imaging response and metastatic rate were analyzed using Prism Graphpad 6. Comparisons between the groups were performed using Pearson’s chi-square test. The Kaplan-Meier method and Pearson’s chi-square test were used to estimate the survival outcomes associated with beta-blocker use using SPSS v22.0 (IBM, Armond, NY, USA). A *p* value of < 0.05 was considered significant.

## 3. Results

### 3.1. Propranolol Enhances Cisplatin and Radiation Sensitivity of PC9 and A549 Tumor Cells In Vitro

**Figure 1:**
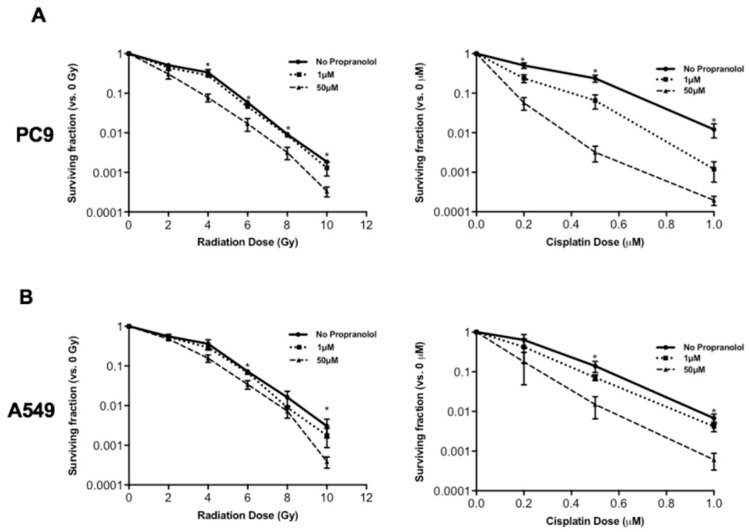
Dose-survival curves of PC9 (**A**) and A549 (**B**) cells treated with varying cisplatin and radiation doses with or without propranolol. Propranolol was administered at 0, 1, or 50 micromolar (μM) to PC9 and A459 cells immediately before cisplatin exposure or irradiation (gray (Gy)). In clonogenic survival assays, propranolol (50 μM) significantly sensitizes tumor cells to cisplatin and radiation when compared to untreated (no propranolol) cells. * *p* < 0.05.

To assess the role of beta-blockers as a therapeutic sensitizer in NSCLC, we analyzed the clonogenic survivability of the PC9 and A549 human NSCLC cells treated with propranolol (non-specific β-adrenergic receptor antagonist) prior to irradiation or cisplatin exposure. Clonogenic assay results of propranolol-treated PC9 cells (Figure 1A) and A549 cells (Figure 1B) are plotted in dose-survival curves. Compared to radiation alone, 50 μM of propranolol decreased clonogenic survival of PC9 cells treated with 4 Gy, 6 Gy, 8 Gy, and 10 Gy (Figure 1A, *p* < 0.05). In A549 cells, 50 μM of propranolol decreased clonogenic survival of cells treated with 6 Gy and 10 Gy (Figure 1B, *p* < 0.05. Propranolol at a lower dose of 1 μM did not have a significant impact of clonogenic survival for either PC9 or A549 cells. Comparing to cisplatin alone, 50 μM of propranolol decreased clonogenic survival of PC9 cells treated with cisplatin at 0.2, 0.5, and 1.0 μM (Figure 1A, *p* < 0.05). In A549 cells, 50μM of propranolol decreased clonogenic survival of cells treated with 0.5 and 1.0 μM of cisplatin (Figure 1B, *p* < 0.05). The decrease in survival fraction in propranolol-treated cells suggests that propranolol sensitizes both PC9 and A549 tumor cells to the direct cytotoxic effects of radiation and cisplatin.

### 3.2. Propranolol Downregulates the β-AR Mediator p-PKA in PC9 and A549 Tumor Cells In Vitro

**Figure 2:**
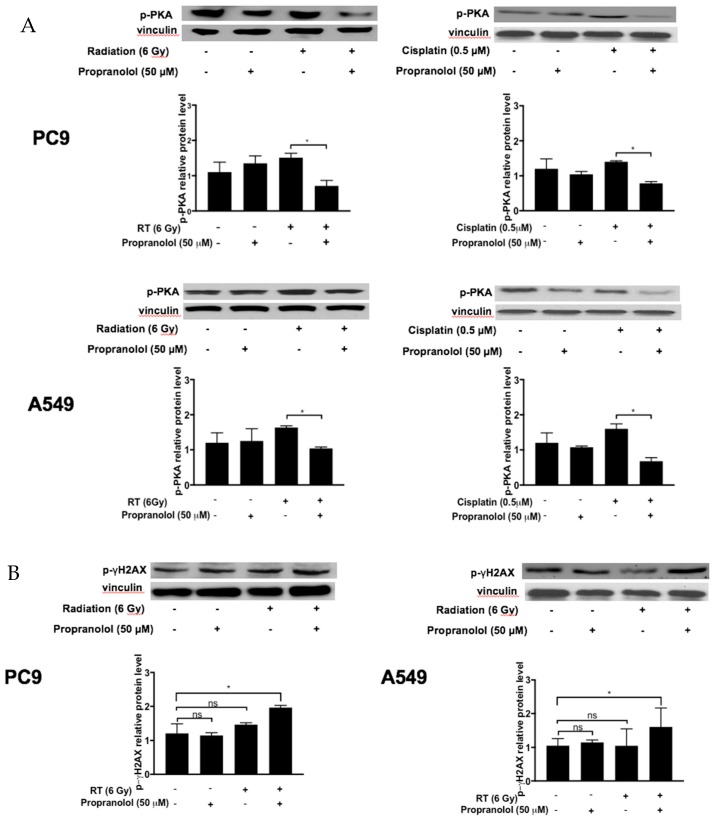
Effect of propranolol on protein kinase A (PKA) and gamma nucleosome core histone H2A (γH2AX) protein expression. PC9 and A549 lung cancer cells were treated with/without propranolol (50 μM) 8 h prior to irradiation (RT) (6 Gy) or cisplatin (0.5 μM) treatment. The protein levels of phospho-PKA (p-PKA) (**A**) and phospho-gamma nucleosome core histone H2A (p-γH2AX) (**B**) were analyzed by Western blot. * *p* < 0.05.

We sought to determine if propranolol can modulate the β-AR pathway in PC9 and A549 lung tumor cells by measuring protein expression of activated phospho-protein kinase A (p-PKA), a downstream mediator in the β-AR pathway. PC9 and A549 cells were treated with radiation (6 Gy) or cisplatin (0.5 μM) after pre-treatment with propranolol (50 μM). Treatment of PC9 cells with propranolol combined with radiation or cisplatin decreased the level of p-PKA at 24 h as compared to irradiation or cisplatin alone, respectively (Figure 2A, *p* < 0.05). Similarly, the combination of propranolol with radiation or with cisplatin decreased p-PKA levels at 24 h in treated A549 cells (Figure 2A, *p* < 0.05). The decrease in the β-AR immediate downstream signaling protein p-PKA suggests that propranolol’s effects in lung cancer cells is to down-regulate β-adrenergic pathway. Interestingly, treatment with propranolol alone did not significantly alter p-PKA expression, suggesting a possible synergistic effect of combined beta-blockers with radiation or cisplatin. 

To determine propranolol’s possible role in radiation-induced DNA damage, we analyzed the levels of phosphorylated-γH2AX (p-γH2AX), a marker for DNA double-stranded break. Radiation alone did not significantly increase p-γH2AX expression at 4 h in both PC9 and A549 cells (Figure 2B). However, pre-treatment with propranolol (50 μM) in combination with radiation increased p-γH2AX expression at 4 h compared to the control PC9 cells and A549 cells (Figure 2B, *p* < 0.05). The increased p-γH2AX expression suggests that propranolol may impair DNA double-stranded strand break repair associated with radiation.

### 3.3. Patient Beta-Blocker Use and Association with Therapy Responses and Patient Outcomes

We retrospectively evaluated 77 patients with Stage IIIA NSCLC who received neoadjuvant chemoradiation followed by surgery. Patient and tumor characteristics are listed in Table 1. The median age of patients was 65 (range 41–79 years). The majority of patients had N2 disease. Induction chemotherapy consisted of a carboplatin or cisplatin doublet. Concurrent radiation consisted of a total dose of 41.4–66.6 Gy, with 51% patients receiving at least 60 Gy to gross disease. Sixteen patients took beta-blockers during the course of treatment for NSCLC, and 61 patients did not. Patients who took beta-blockers were more likely to take aspirin (*p* = 0.02) and ACEi (*p* < 0.01). Other factors such as age, tumor characteristics, and radiation dose received were not significantly different between the groups. Median follow-up was 4.8 years (range, 1–114 months).

**Figure 3:**
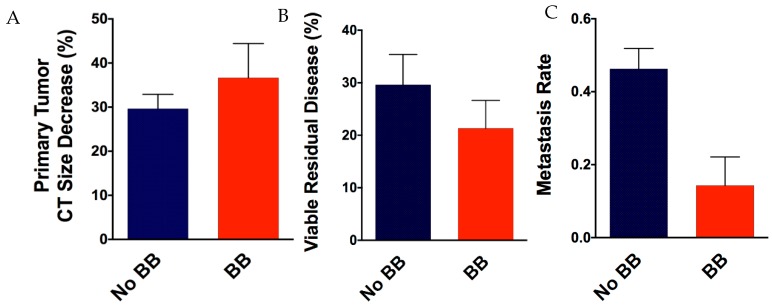
Local primary tumor response and metastases rate. Primary tumor response was assessed by comparing the largest diameter of the lung tumor on baseline computerized tomography (CT) scans with that on CT scans 4–6 weeks after chemoradiation (**A**). Pathological response was determined as percent viable residual tumor at time of surgery (**B**). Metastases rate was determined by the incidence of distant metastatic disease (**C**). BB, beta-blocker.

**Figure 4:**
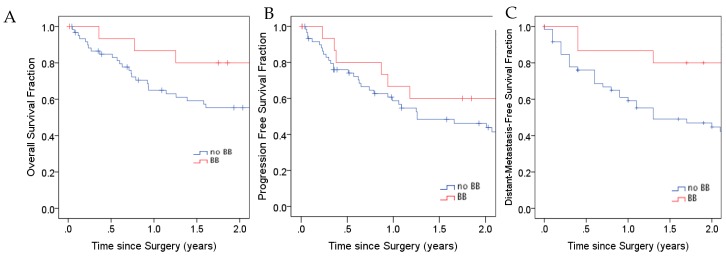
The effect of beta-blockers on patient clinical outcomes. Comparison of overall survival (OS), progression-free survival (PFS), and distant-metastasis-free survival (DMFS) between patients who took beta-blockers during treatment for non-small cell lung cancer (NSCLC) and those who did not. BB, beta-blocker.

Given that β-AR blockade sensitizes lung tumor cells in vitro to cisplatin and radiation, we sought to determine if beta-blocker use affected primary lung tumors response. There was a trend associated with greater primary tumor response by CT imaging (Figure 3A) and beta-blocker use, as mean reduction in largest lung tumor diameter was 37% in the beta-blocker group compared to no beta-blocker group (mean decrease 30%, *p* = 0.36). We also observed a trend in reduced pathological residual disease (Figure 3B) in the beta-blocker group (mean residual viable disease 21%) compared to the no beta-blocker group (mean residual viable disease 30%, *p* = 0.43). Noticeably, analyses showed a significantly decreased incidence of distant metastasis (Figure 3C) in patients who took beta-blockers (13%) compared to those who did not (43%), (risk ratio of 0.19; *p* = 0.03). The Kaplan–Meier curves of overall survival (OS), progression-free survival (PFS), and distant metastasis-free survival (DMFS) in the two groups of patients are shown in Figure 4. Beta-blocker use was associated with a trend to improved OS at 1 year (81.3% vs. 57.4%, *p* = 0.08) and a trend to improved median DMFS (2.6 years vs. 1.3 years, *p* = 0.16).

## 4. Discussion

To our knowledge, this is the first study investigating beta-blockade as a chemotherapy and radiation sensitizer in human lung cancer cell lines. Our clonogenic survival assay suggested that β-AR blockade with propranolol enhances cisplatin and radiation sensitivity of human NSCLC cancer cells in vitro. Our results support previous observation of the sensitizing effect of propranolol in human gastric adenocarcinoma cell lines treated with radiation [25] and in pancreatic tumor models treated with chemotherapy in a stress-induced environment [19]. Previous studies have indicated that β-AR blockage in combination with irradiation or cisplatin results in increased apoptosis in tumors through down-regulation of the apoptosis suppressor Bcl-2 and up-regulation of pro-apoptotic p53 family target genes Bax, caspase-3, and caspase-9 [14,16,19,25,32,33]. As apoptosis is one mode of radiation- and cisplatin-induced cell death, these findings suggest that β-AR blockade with beta-blockers may enhance cell killing and increase the therapeutic effect of chemoradiation.

In addition, we demonstrate that β-adrenergic blockade with propranolol in addition to irradiation or cisplatin may down-regulate the cyclic adenosine monophosphate (cAMP)-dependent PKA, a downstream target of the β-adrenergic receptor. Interestingly, our experiments suggested that the reduction in p-PKA was a consequence of the synergistic effect between beta-blocker and radiation or cisplatin, as single modality therapy with propranolol or either therapeutic treatment had no effect on p-PKA. Down-regulation of p-PKA may result in decreased signaling in multiple downstream pathways including the Akt, Erk1/2, and NFκB, which affect tumor proliferation, differentiation, angiogenesis, survival and migration [23,34,35,36]. More importantly, it has been shown that p-PKA inactivates the pro-apoptotic protein, Bcl-2-associated death promoter (BAD), by phosphorylation. As previously mentioned, studies have indicated that β-AR blockage in combination with irradiation or cisplatin results in increased apoptosis in tumors. One might hypothesize that this pro-apoptotic effect is achieved through less p-PKA inactivation of BAD. Together with our results, these studies suggest the beta-blocker’s down-regulation of PKA activation may enhance the chemo- and radio-sensitivity of lung tumor cells by promoting tumor killing and reducing tumor growth.

Another possible explanation of the sensitizing effect of beta-blocker may be its direct impact on DNA double-stranded break repair. When cells were treated with radiation alone, there was no significant increase in p-γH2AX at 4 h, suggesting that double strand repair may have taken place. As illustrated in our study, the addition of propranolol to radiation increased p-γH2AX expression. As detection of p-γH2AX indicates the presence of DNA double-stranded break, this finding may indicate delayed repair of radiation-induced double-stranded DNA break, leading to increased cell death. This speculation warrants further analysis of DNA damage repair-related proteins to collaborate our findings. Overall, the β-adrenergic pathway may regulate multiple processes that contribute to tumor therapeutic resistance and disease progression, and beta-blockers may modulate tumor treatment response via multiple intracellular pathways.

Repurposing existing well-characterized non-cancer drugs has been shown to be an efficient, cost-effective, and low toxicity route to identifying new cancer therapeutics [37]. This analysis is the first attempt to evaluate beta-blocker effect in a select cohort of stage IIIA NSCLC patients treated with trimodality therapy. Our findings are in accordance with the findings of MD Anderson retrospective clinical reviews of beta-blockers in NSCLC. In a cohort of 722 patients with NSCLC treated with definitive radiation therapy, Wang et al. identified that incidental beta-blocker use was associated with improved OS, DMFS, and disease-free survival (DFS) [29,30]. Our analysis of stage IIIA NSCLC patients with concurrent beta-blocker use also demonstrated an association with significant reduction in metastatic rate and a trend to improved median DMFS and median OS. Our review was limited by the smaller cohort size as compared to the MD Anderson study, which may have limited the study’s power to detect significant differences in OS and DMFS. Experiments in colon carcinoma cell line showed that β-AR signals through phospholipase C and protein kinase C and membrane metalloproteinases to induce cellular migration [23,38,39]. These findings demonstrate that β adrenergic signaling affects tumor metastatic potential, an effect that can be reversed by β-AR blockade [20,21]. As observed in clinical studies, these pre-clinical results suggest a possible synergistic therapeutic effect in reduction of distant metastases and a possible OS and DMFS advantage in NSCLC patients who use beta-blockers.

Given that β-AR signaling inhibition has been demonstrated to decrease mitogenic signaling and enhance apoptosis [19,23,24,25], coupled with the results of our in vitro assays demonstrating the therapeutic sensitizing effect of propranolol, we expected a greater local tumor response in patients who took beta-blockers. Although there was a trend to improved tumor response, we did not detect a statistically significant difference in primary tumor treatment response on imaging or by pathology in our patients who took beta-blockers. Similarly, the MD Anderson analyses found no association between beta-blocker use and locoregional progression-free survival. One possible explanation is that beta-blockers exert their tumor control effect mainly through the inhibition of tumor metastasis. Beta-blockers have been shown to suppress tumor cell migration and enhance chemotherapy sensitivity, which may be more important for killing microscopic tumor foci. However, the exact mechanism through which beta-blockers lead to decreased metastases has yet to be defined given the drug’s pleomorphic tumor effects. In addition, we suspect different tumor types may have differential sensitivity towards beta-adrenergic inhibition. The synergistic effect observed with the two adenocarcinoma cell lines may not be detected in our patient study population, which was treated for all types of NSCLC, including squamous cell carcinoma and adenocarcinoma. In addition, the in vitro effect of propranolol may not be readily translated to the biological effect of beta-blockers on tumors in actual patients. 

In addition to the study being a retrospective review at a single institution, there are a few limitations in this study. Given the relatively small number of patients who were using beta-blockers during their NSCLC treatment, it was not feasible to subdivide and evaluate the effect of non-selective agents versus β-1 selective agents on tumor control. Preclinical studies have shown that non-selective agents and β-selective agents may differentially affect tumor proliferation and survival in human cancer cell lines [16,40,41]. Our analysis was based on all beta-blocker use, and we may fail to detect a significant benefit of one class of beta-blocker on tumor control. In addition, due to the relatively small cohort of patients reviewed for the study, it was not feasible to conduct a multivariate analysis on the association of beta-blocker intake and patient survival outcome. Age, Karnofsky performance score, tumor histology, radiation dose, gross tumor volume, and other negative prognostic factors may need to be adjusted to remove any confounding influence on beta-blocker and tumor control association. Given the small number of patients included in this retrospective study, an evaluation of larger clinical cohorts is indicated.

This study is the first to report both cisplatin and radiation sensitizing effect of beta-blockers in NSCLC in vitro. This promising finding, coupled with results of the retrospective clinical review of stage IIIA NSCLC patients treated with induction chemoradiation at our institution, suggest that beta-blocker use may improve NSCLC outcomes at a relatively low cost, both in financial terms and with respect to toxicity. Additional experiments to investigate the mechanisms of β-AR blockade in lung cancer treatment sensitization are warranted. Larger clinical studies examining the effect of beta-blocker in locally advanced NSCLC patients without hypertension or heart conditions may prove valuable. The aggregated results would reveal new insights about treatment-resistance and offer new therapeutic options for locally advanced NSCLC.

## Figures and Tables

**Table 1:** Patient and tumor characteristics. NSCLC: non-small cell lung cancer; ACE: angiotensin-converting enzyme.

Characteristics	Number of Patients (%) Beta-Blockers (*n* = 16)	Number of Patients (%) No Beta-Blockers (*n* = 61)	*p* Value
Sex			0.99
Female	6 (38)	23 (38)	
Male	10 (62)	38 (62)	
Age, years			0.29
<65	5 (31)	28 (46)	
≥65	11 (69)	33 (54)	
T Category			0.24
T1,2	10 (63)	47 (77)	
T3,4	6 (38)	14 (23)	
N Category			0.32
N0,1	3 (19)	6 (10)	
N2	13 (81)	55 (90)	
Tumor Histology			
Squamous cell	6 (38)	13 (21)	0.18
Adenocarcinoma	6 (38)	31 (51)	
NSCLC, not otherwise specified (NOS)	4 (25)	17(28)	
Radiation Dose, Gy			0.70
<60	7 (54)	23 (48)	
≥60	6 (46)	25 (52)	
Aspirin			0.02
No	10 (62)	53 (87)	
Yes	6 (38)	8 (13)	
ACE inhibitor			<0.01
No	11 (69)	57 (93)	
Yes	5 (31)	4 (7)	
